# Non-linear and spectral EEG dynamics as stage–specific biomarkers of neuropsychological recovery post-stroke

**DOI:** 10.3389/fnins.2026.1862806

**Published:** 2026-08-13

**Authors:** Larisa Mayorova, Anastasia Kushnir, Alexey Yakovlev, Andrey Grechko, Galina Portnova

**Affiliations:** 1 Institute of Higher Nervous Activity and Neurophysiology RAS, Moscow, Russia; 2 Laboratory of Experimental Neurology and Neuroimaging, Federal Research and Clinical Center of Intensive Care Medicine and Rehabilitology, Moscow, Russia

**Keywords:** cognitive function, fractal dimension, post-stroke recovery, power spectral density, stroke

## Abstract

**Introduction:**

Resting-state electroencephalography (EEG) offers promising window into the neuroplastic changes underlying recovery after stroke. This longitudinal study aimed to characterize the stage-specific dynamics of non-linear (fractal dimension, FD) and linear (power spectral density, PSD) EEG metrics and to evaluate their associations with recovery of neuropsychological functions.

**Methods:**

We recruited 44 patients with left-hemisphere ischemic stroke at various recovery stages and 25 age-matched healthy controls. All participants underwent high-density EEG recording at two time points (~5–7 weeks apart). The EEG was analyzed for FD, PSD across standard frequency bands.

**Results:**

A rehabilitation-related increase in FD in the right hemisphere was observed in all patient groups except those in the early recovery stage, where a unique decrease in left-hemisphere FD occurred. This right-hemispheric FD increase correlated with improvements in motor control function. Spectral changes were also stage-specific: a decrease in frontal beta power (14–20 Hz) was linked to longer inter-session intervals, while a right posterior increase in slow-wave activity (2–6 Hz) correlated with gains in auditory-verbal memory. Conversely, a reduction in alpha power (7–9 Hz) was associated with improved visuospatial ability and motor functions, but only in early and intermediate recovery stages. Recovery after stroke is subserved by dynamic, phase-specific neurophysiological processes reflected in the FD and spectral composition of spontaneous brain activity. The right hemisphere appears to play a crucial compensatory role, with FD serving as a sensitive biomarker of motor planning recovery.

**Conclusion:**

These findings underscore the necessity of stage-specific neurorehabilitation framework, informed by objective EEG biomarkers, to individualize and enhance therapeutic efficacy.

## 1 Introduction

Stroke remains a pressing medical and social concern. According to the World Stroke Organization, over 12 million first-time strokes occur globally each year, with approximately 6.5 million proving fatal. Current estimates indicate that more than 100 million people worldwide are living with the consequences of a stroke. Although stroke risk increases predictably with age, more than 60% of all strokes affect individuals under the age of 70, and one in six strokes (16%) occurs in people under 50 years old (World Stroke Organization, 1234). Return to pre-stroke employment is possible for only 15% of individuals (Kadykov et al., 2022). The majority of patients require ongoing medical supervision (Johnson et al., 2019; Krishnamurthi et al., 2020).

Cognitive decline, alongside motor deficits, significantly impacts the quality of life in post-stroke patients (El Husseini et al., 2023). The presence of cognitive impairment (CI) is considered a key risk factor for mortality within 5 years after a stroke (Gaynor et al., 2018).

Existing research findings contribute to the understanding of the dynamic trajectory of functional recovery post-stroke, which is crucial in clinical practice for planning rehabilitation strategies and predicting patient outcomes (Ostrova et al., 2019). However, the dynamics of neuropsychological functions in patients at different time points post-stroke require further comprehensive investigation. Furthermore, data on the dynamics of functional brain reorganization throughout patient recovery are insufficient. Questions of particular practical and theoretical interest include: (1) What is the predictive capacity of early-stage brain activity for long-term functional recovery? (2) How does brain activity change during early stages compared to later stages of recovery? (3) What is the localization of specific brain reorganization patterns at different stages of post-stroke recovery? (4) Which metrics of instrumentally measured brain activity may prove most sensitive for clinical purposes?. Therefore, the objective of our research was to assess the dynamics of functions and brain activity in patients at various stages following an ischemic stroke.

Electroencephalography (EEG) is a simple, readily available diagnostic method with excellent temporal resolution for assessing changes in brain activity. This is often a crucial advantage for post-stroke patients, as other examination methods may be unsuitable for regular monitoring. EEG becomes particularly valuable when clinical tests are ineffective and fail to show significant differences, as it can reveal predictive neural biomarkers of recovery. Research confirms that key prognostic markers are found in the spatial and spectral patterns of functional connectivity. For instance, a longitudinal study confirmed two common recovery patterns—proportional recovery (PROP) and poor recovery (POOR)—and found that PROP patients initially had greater functional connectivity, especially between key motor areas, and better preserved corticospinal tract integrity. Critically, these connections degraded over time in POOR patients but not in PROP patients (Guggisberg et al., 2017).

The spectral characteristics of this connectivity are highly informative. Alpha-band (8–13 Hz) functional connectivity, particularly between the motor cortex and parietal or contralateral frontotemporal areas, is strongly associated with greater brain excitability following therapeutic interventions like and can predict response to such neuromodulation (Hordacre et al., 2018). Furthermore, stronger functional connectivity within the alpha and beta bands in the affected hemisphere at 4 weeks post-stroke can predict upper limb function at 8 weeks, highlighting its potential as a recovery biomarker (Hoshino et al., 2020). The dynamic nature of alpha activity is also crucial; motor recovery is linked to alpha desynchronization in the motor cortex, with the optimal direction of change (increase or decrease) depending on the patient's initial level, making it a key marker for stratification (Ray et al., 2020).

Recovery is also reflected in how brain regions communicate during specific tasks. Post-intervention, changes in inter-hemispheric coupling—such as a reduction in gamma-band communication from the ipsilesional to the contralesional motor cortex during movement preparation—are linked to more normalized brain activity during execution (Wilkins et al., 2020). Specific phase synchrony indices offer additional biomarkers: reduced alpha-band synchrony between motor cortices correlates with initial impairment, while elevated theta-band synchrony centered on the contralesional hemisphere correlates with recovery gain (Kawano et al., 2020). Finally, cross-frequency interactions, such as pre-frontomotor delta-beta coupling, are altered after stroke, with higher initial coupling associated with better motor function and recovery, presenting a complex but promising biomarker (Mark et al., 2024).

Alongside these connectivity measures, other established EEG biomarkers for rehabilitation monitoring include power spectral density (PSD) in the alpha band, which effectively differentiates between healthy and affected hand movements (Setiawan et al., 2019). The revised brain symmetry index serves as a prognostic measure, where its negative correlation with motor improvement suggests it can predict outcomes (Mane et al., 2019). Furthermore, relative beta power in frontal and central regions is linked to mental fatigue during Motor Imagery Brain-Computer Interface therapy (Foong et al., 2020), underscoring the need for adaptive brain-computer interface (BCI) strategies that update subject-specific models to enhance system efficacy for rehabilitation (Ang and Guan, 2017).

While significant progress has been made in identifying linear EEG biomarkers for motor recovery after stroke, such as alpha power, frequency-specific functional connectivity, and symmetry indices, critical gaps persist, limiting a more holistic and predictive understanding of rehabilitation outcomes. A primary shortcoming is the underutilization of non-linear EEG analysis, which may better capture the brain's inherent complexity and dynamic adaptability. Current biomarker research relies heavily on methods such as spectral power and phase synchrony, which often assume stationary, linear relationships. In contrast, the post-stroke brain is a non-stationary system undergoing profound reorganization. Metrics of signal complexity, entropy, and fractal dynamics could provide valuable insight into the system's information-processing capacity and adaptive potential, potentially distinguishing between rigid, maladaptive states and flexible, recovering network configurations (Castellanos et al., 2012). An equally consequential gap is the dissociation between motor-network biomarkers and the cognitive and affective domains that fundamentally influence rehabilitation engagement and success. Existing literature focuses intensely on the motor cortex and its direct connections, yet recovery in clinical practice is inseparable from a patient's attentional capacity, motivation, executive function, and emotional state. For instance, frontal theta oscillations as a marker of cognitive control and working memory could be used as a valuable indicator of cognitive engagement during motor network activation in therapy (Ulanov and Shtyrov, 2022). Similarly, the role of the reward network, measurable through event-related potentials, remains largely overlooked as a modulator of motor learning (Palidis et al., 2019). Although EEG correlates of affective states such as post-stroke depression and apathy—including frontal alpha asymmetry—are well documented, they are seldom integrated with motor connectivity measures to form a composite prognostic profile (Marques et al., 2024).

This study is one of the few to employ an extensive neuropsychological test battery, providing a meticulous assessment of various aspects of speech function, as well as gnosis, praxis, and memory. We focused on identifying biomarkers associated with neuroplasticity mechanisms, which is crucial for developing novel neurorehabilitation methods based on targeted brain stimulation and for refining standard restorative therapies. Patients with left-hemisphere ischemic stroke were specifically recruited for this study to ensure a homogeneous cohort with respect to both lesion topography and functional deficits. Given that over 95% of right-handed individuals exhibit left-hemisphere language dominance (Knecht et al., 2000; Springer et al., 1999), restricting inclusion to left-sided strokes allowed for consistent assessment of speech and language recovery, which was the primary clinical focus of the rehabilitation center. Achieving these goals necessitates a comprehensive, interdisciplinary approach that integrates brain imaging techniques with behavioral assessment methods in clinical practice.

## 2 Methods

### 2.1 Participants

#### 2.1.1 Patient group

The study enrolled patients admitted to the Center for Speech Pathology and Neurorehabilitation (Moscow) both in the early recovery period following a stroke and at its later stages. Patients were hospitalized for a period of 45 to 90 days with the aim of undergoing speech rehabilitation therapy. In cases where patients presented with a non-isolated speech function impairment, i.e., one occurring in combination with other higher mental or motor function deficits, the therapeutic intervention was also directed at correcting these comorbid conditions.

Inpatients of both sexes, aged 35–80, were selected for the study based on the following criteria: (1) first-ever ischemic stroke in the territory of the left middle cerebral artery or left anterior cerebral artery; (2) stroke onset 3 months prior; (3) confirmation by brain MRI; (4) documented right-handedness; and (5) moderately severe activity limitation in daily living (61–80 points on the Barthel Index).

Patients were excluded from the study if they had: (1) hemorrhagic stroke; (2) transient ischemic attack; (3) brain tumor; (4) head trauma; (5) epilepsy; (6) psychiatric disorders; (7) inflammatory brain lesions; (8) decompensated somatic diseases; (9) malignant neoplasms; or (10) any condition that, in the investigator's opinion, could impede study participation.

Participants could be withdrawn from the study for: (1) screening failure; (2) erroneous enrollment of an ineligible patient; (3) refusal or inability to comply with the protocol; (4) patient's decision to withdraw for any reason; (5) pregnancy; or (6) any unanticipated circumstance where the investigator believed continued participation would be harmful. Prior to enrollment, all patients provided written informed consent.

The mean age of the enrolled patients was 60 [interquartile range (IQR):14] years, ranging from 44 to 71 years. Males constituted 53.1% of the cohort, and females 46.9% (see Table 1). Twenty-nine patients (57.1%) had a higher education degree; the remaining patients had secondary specialized education.

**Table 1:** Demographic characteristics of the groups of subjects.

Factor	Control group			Patients group			***T*** **-Test**	
	Mean	Median	SD	Mean	Median	SD	t	p
Age	61.12	61	10.79	59.39	60	9.71	0.68	0.5
dTime (days)	37.3	33	16.8	36.14	32	22.14	0.25	0.8
BMI	26.18	26.95	2.78	28.45	29	4.84	−2.14	0.03
**Sex**	**Women**	**Men**	**Women**		**Men**		**Chi–square test**	
					χ^2^	**p**
	10	15	19		25		0,07	0,8

SD, standard deviation; dTime, time interval (in days) between the first and second EEG recording sessions; BMI, body mass index (kg/m^2^); t, independent samples *t*-test statistic; χ^2^, chi-square test statistic; p, significance level. Age, BMI and dTime were analyzed using independent samples *t*-tests as they were normal according to Shapiro–Wilk.

The diagnosis of cerebral infarction was established based on the diagnostic criteria of the International Classification of Diseases, 10th Revision (World Health Organization, 2004). Ischemic stroke subtypes were classified according to the Trial of Org 10172 in Acute Stroke Treatment criteria (Chung et al., 2014). The overwhelming majority of patients presented with the atherothrombotic subtype of ischemic stroke as the leading pathogenetic variant, localized in the left middle cerebral artery territory.

Comorbidity was highly prevalent. Cardiovascular diseases, nervous system disorders, and nutritional/metabolic diseases were observed in over 30% of patients. Musculoskeletal/connective tissue disorders, gastrointestinal diseases, and renal/urinary tract diseases had a prevalence of up to 13%. Hepatobiliary and respiratory/thoracic/mediastinal diseases were less frequent (up to 10%). Chronic infectious diseases, along with disorders of the hearing, reproductive/mammary, and endocrine systems, were rare, with a prevalence of up to 5%.

All patients received standard pharmacological therapy, including antiplatelet/antithrombotic agents, lipid-lowering drugs, renin-angiotensin system agents, β-blockers, diuretics, calcium channel blockers, and antidiabetic medications. Other pharmacological groups were used less frequently.

Neurological examination revealed moderate neurological deficit, manifested as paresis, hypoesthesia, and ataxia. Patients demonstrated moderate independence in daily living activities, corresponding to a Barthel Index score of 66.2 ± 5.3 points, indicating the ability to perform basic self-care tasks but requiring occasional assistance.

All patients were divided into subgroups according to recovery stage. Data are presented in Table 2.

**Table 2:** Clinical subgroups of patients stratified by recovery stage.

Recovery stage	Valid N	dTime (days)		Days after stroke	
		Mean	Std. dev.	Mean	Std. dev.
Subacute stage	11	33.91	13.71	78.27	18.53
Early recovery	12	33.17	17.20	133.92	19.42
Intermediate recovery	14	42.21	31.94	250.29	28.77
Ongoing recovery	7	29.57	10.95	431.71	85.94

Abbreviations: Valid N, number of patients in each subgroup; dTime, time interval (in days) between the first and second EEG recording sessions; SD, standard deviation.

#### 2.1.2 Control group

The study included healthy elderly volunteers as a control group (Table 1). Participants in this group had no significant vascular or neurological diseases (e.g., cerebrovascular disease, dyscirculatory encephalopathy, stroke regardless of location, or stage II or higher hypertension). Isolated, non-systemic episodes of elevated blood pressure and early manifestations of atherosclerosis were permitted. The control group had no history of psychiatric or other neurological disorders (including traumatic brain injury and epilepsy).

The EEG study was conducted twice on the same equipment at the Institute of Higher Nervous Activity and Neurophysiology, Russian Academy of Sciences, with an interval of 22 to 80 days (consistent with the main patient cohort). The experimental procedure was designed to closely mirror that used for the patient groups.

### 2.2 Experimental paradigm

The EEG recordings were performed by a single specialist in a dedicated EEG laboratory. During the recording, patients were instructed to remain relaxed and avoid focused mental activity. The procedure duration did not exceed 30 min. All recordings were conducted in the morning. Prior to the procedure, patients were asked to be well-rested and, if they were smokers, to refrain from smoking for at least 2 h. EEG recordings and neuropsychological assessments were conducted as separate procedures at two matched time points: upon admission (1st session) and upon discharge (2nd session), approximately 5–7 weeks apart. EEG was recorded during a resting-state condition with eyes closed, not during neuropsychological testing. For analysis, we selected continuous EEG fragments with eyes closed lasting longer than 150 s (mean 181.5 ± 11.7 s), free of artifacts. Neuropsychological assessments were performed on separate days within the same time window to avoid interference with EEG recordings. Between these sessions, standard rehabilitation was carried out, including measures aimed at speech and motor recovery.

### 2.3 EEG registration

The EEG was acquired using a 19-channel EEG amplifier Encephalan (Medicom MTD, Taganrog, Russia). The sampling rate was 250 Hz. The amplifier band-pass filter was nominally set to 0.05–70 Hz. AgCl electrodes were placed according to the International 10–20 system. The electrodes placed on the left and right mastoids served as joint references under unipolar montage. The vertical EOG was recorded with AgCl cup electrodes placed 1 cm above and below the left eye, and the horizontal EOG was acquired by electrodes placed 1 cm lateral from the outer canthi of both eyes. The electrode impedances were kept below 10 kΩ.

Continuous EEG corresponding to the resting state of each subject was cleaned from eye movements by an ICA-based algorithm in the EEGLAB plugin for MATLAB 2022 (Mathworks Inc., Natick, MA, USA). Muscle artifacts were cut out through manual data inspection. The continuous resting-state EEG of each subject was initially filtered with a 0.5–30 Hz band-pass filter using the EEG recording software. Subsequent pre-processing was performed with a 1.6–30 Hz bandpass filter using EEGLAB's finite impulse response (FIR) filter (pop_eegfiltnew). The filter order was estimated automatically by the function based on the sampling rate and the desired transition bandwidth.

We recruited continues EEG fragments with eyes closed with duration longer 150 sec (mean 181.5 ± 11.7). Only resting-state EEG fragments with eyes closed were used for analysis. No EEG data were collected during the neuropsychological testing procedures. Linear and non-linear EEG metrics were calculated, such as power spectral density (PSD), fractal Dimension (FD) and peak alpha frequency (PAF). PSD was computed using MATLAB 2022 (Mathworks Inc., Natick, MA, USA). Spectral estimation was performed via Fast Fourier Transform (FFT), with subsequent normalization of the EEG spectra for each auditory stimulus. Normalized power was integrated within standard frequency bands: delta (2–4 Hz), theta1 (4–6 Hz), theta2 (6–8 Hz), alpha1 (8–10 Hz), alpha2 (10–12 Hz), alpha3 (12–14 Hz), beta (14–20 Hz). FD, quantifying signal complexity, was computed using a custom C++ implementation of the Higuchi algorithm [24]. The method involves: Generating k decimated time series (k-sampling interval). Computing curve lengths [*L*(*k*)] as normalized sums of absolute differences. Determining FD via linear regression of loglog *L*(*k*)vs. $\log\log\;\frac{1}{k}\;$. For fractal dimension (FD) computation, the pre-filtered data were additionally filtered using a 4th-order Butterworth bandpass filter (1.6–30 Hz). This step was applied sequentially—after the initial 0.5–30 Hz pre-processing filter—to restrict the frequency range for FD estimation and minimize the influence of residual low-frequency fluctuations. The use of two distinct filtering steps reflects our aim to balance standard EEG pre-processing practices with the specific frequency-band requirements of fractal dimension analysis, which is known to be sensitive to low-frequency drift.

PAF was calculated by analyzing the power spectra of the scalp and deep electrodes by FFT as the frequency of the center of gravity in the range of 8–12 Hz to exclude PAF lying outside the fixed frequency bands (Haegens et al., 2014) in patients in the state of general anesthesia. The frequency of the center of gravity is defined as “the weighted sum of spectral evaluations divided by the power” of the α rhythm:


$$\begin{array}{l} PAF=\frac{\sum \left(a\left(f\right)\times f\right)}{\left(f\;\right)}, \end{array}$$

where a(*f*) is the spectral power at frequency *f* in the α band.

### 2.4 Neurological and neuropsychological assessment

To assess the severity of focal cognitive impairment in patients who had suffered an ischemic stroke, neurological and neuropsychological examinations were conducted before the start of the treatment and rehabilitation cycle, as well as after its completion. The neurological examination included the National Institutes of Health Stroke Scale (Brott et al., 1989) and the Barthel Index (Mahoney and Barthel, 1965). Neuropsychological examination was conducted according to the “10-point assessment of mental functions” methodology (Shklovskii and Repin, 2016). Table 3 shows the average values obtained during neurological and neuropsychological assessments. A detailed description of all scales and their scoring is provided in Table 4

**Table 3:** Neurological and neuropsychological assessment results at first and second assessment sessions.

Parameters	1st assessment				2nd assessment				Wilcoxon Matched Pairs Test		
	Mean	Median	Range	Quartile	Mean	Median	Range	Quartile	*Z*	*p*	*r*
NIHSS	7.16	6	11	3.5	6.88	6	11	3	2.80	0.005	0.40
Barthel	80.11	85	40	10	80.81	85	40	10	2.20	0.03	0.31
Receptive language	7	7.7	8.2	4.3	7.79	8.3	7.2	3.7	5.30	< 0.001	0.75
Expressive language	5.45	5.5	10	6.3	6.22	6.6	10	6.20	5.33	< 0.001	0.75
Motor function	8.3	9.5	10	2.75	8.6	10	9.5	1.50	3.82	< 0.001	0.54
Motor control function	7.8	8.84	10	3	8.24	9.33	8.33	2.17	4.54	< 0.001	0.64
Visuospatial ability	8.68	10	10	1	9	10	10	1.00	2.93	0.003	0.41
Perceptive	8.94	10	6.5	0.5	9.23	10	5.5	0.00	2.37	0.02	0.34
Memory	5.36	6	9.5	5	6.15	7.25	10	4.13	5.30	< 0.001	0.75
Speech impairment	4.48	4	5	3	4.05	4	5	2.00	3.82	< 0.001	0.54
Motor and cognitive impairment	8.12	8.59	7.4	1.56	8.52	8.97	5.96	1.42	5.78	< 0.001	0.82

SD, standard deviation; NIHSS, National Institutes of Health Stroke Scale (0–31, higher scores indicate greater neurological impairment); Barthel, Barthel Index of activities of daily living (0–100, higher scores indicate greater independence); Z, Wilcoxon matched pairs test statistic; p, significance level; r, effect size calculated as |Z| / √N (small ≥ 0.1, medium ≥ 0.3, large ≥ 0.5).

**Table 4:** Summary of neurological and neuropsychological assessment instruments.

Assessment domain	Test/Scale	Description	Score range
Neurological status	NIHSS (Brott et al., 1989)	Assesses level of alertness, orientation, ability to follow instructions, eye movements, visual fields, facial nerve function, limb movement, sensitivity, ataxia, aphasia, dysarthria, and neglect. Items scored on 3-, 4-, or 5-point Likert scales.	0–31 (higher = worse); 0 = no changes, 31 = death
Functional independence	Barthel Index (Mahoney and Barthel, 1965)	Assesses 10 daily activities: eating, bathing, hygiene, dressing, defecation, urination, rising from bed, walking, climbing stairs. Items scored on 2-, 3-, or 4-point scales with weights from 0 to 20 points.	0–100 (higher = better); 0 = maximum disability, 100 = complete independence
Receptive language (verbal function)	Word comprehension, instruction understanding, grammatical constructions	Understanding words, simple/complex instructions, inflectional, pre-positional, passive, inverted, temporal, genitive, and comparative constructions; grammatical correctness assessment.	0–10 (higher = better)
Expressive language (verbal function)	Dialogical speech, automated speech, repetition, naming, phrase construction	Counting, naming days of week, finishing proverbs; repetition of sounds/syllables/words/series; naming objects/actions; composing phrases from pictures or with given words.	0–10 (higher = better)
Auditory-verbal memory (verbal function)	10-word recall test	Immediate and delayed recall of 10 words.	0–10 (higher = better)
Motor function (non-verbal function)	Motor Hand Exam	Repetition of individual gestures and poses.	0–10 (higher = better)
Motor control function (non-verbal function)	Kinetic praxis	Repeating sequences of gestures; drawing graphic patterns (repeating combinations of rectangular and triangular elements without lifting pen from paper).	0–10 (higher = better)
Visuospatial ability (non-verbal function)	Kohs Blocks	Block design assembly task.	0–10 (higher = better)
Perception (gnosis) non-verbal function)	Object and letter recognition tests	Recognition of geometric shapes, realistic images, crossed-out images, superimposed contours, letters (isolated and superimposed), digits, and numbers.	0–10 (higher = better)

All neuropsychological tests were scored using an 11-point Likert scale (0–10) according to the “10-point assessment of mental functions” methodology (Shklovskii and Repin, 2016). Verbal function tests (receptive language, expressive language, auditory-verbal memory) assess the severity of aphasia and dysarthria; non-verbal function tests (motor function, motor control, visuospatial ability, perception) assess the severity of apraxia and agnosia. For enlarged test categories, average scores were used.

### 2.5 Conventional neurorehabilitation therapy

The neurorehabilitation course included kinesitherapy techniques and intensive speech rehabilitation therapy. Speech therapy was based on the location and extent of the lesion in the hemisphere, the initial severity of speech disorders, the duration of the disease, the form of aphasia, and the level of linguistic disorders (phonetic, lexical, semantic, discursive), as well as the profile of lateral organization of mental functions. These methods, based on the principle of restructuring functional systems, are considered a leading approach in the rehabilitation of patients with organic brain lesions (Shklovskii and Repin, 2016). Speech therapy was based on the location and extent of the lesion in the hemisphere, the initial severity of speech disorders, the duration of the disease, the form of aphasia, and the level of linguistic disorders (phonetic, lexical, semantic, discursive), as well as the profile of lateral organization of mental functions.

Throughout the study, patients received standardized basic therapy aimed at treating the underlying disease and providing secondary stroke prevention, combined with multimodal rehabilitation stimulation.

The highly uniform pharmacological regimen included antihypertensive, antithrombotic, and antiarrhythmic agents. Correction of dyslipidemia and disorders of carbohydrate metabolism was performed when clinically indicated.

The multimodal rehabilitation program (the duration of one course was approximately 4.7 weeks) comprised daily sessions in music and computer classes, occupational therapy, intensive speech therapy (individual sessions with a speech therapist twice daily for 35 min each), individual sessions with a neuropsychologist (45–60 min each), therapeutic physical exercise, massage, positional therapy for paretic limbs, mechanotherapy, and cardiotraining. The average total daily duration of rehabilitation techniques per patient was approximately 4–4.5 h.

### 2.6 Statistical analysis

Statistical analysis was performed using Statistica 12 software (StatSoft). A repeated-measures analysis of variance (ANOVA) was conducted for parameters FD, PAF, and PSD, with the following within-subject factors: lateralization (two levels: left and right hemispheres), Period (two levels: 1st session and 2nd session), and Frequency (seven levels: delta, theta1, theta2, alpha1, alpha2, alpha3, beta), and a between-subject factor of Group (five levels: subacute stage, early recovery, intermediate recovery, ongoing recovery, and control). Electrodes were grouped into regions of interest: frontal (F3, Fz, F4), central (C3, Cz, C4), parietal (P3, Pz, P4), and occipital (O1, O2). To assess lateralization differences, we compared right-hemisphere electrodes (F4, C4, O2, P4) with their left-hemisphere counterparts (F3, C3, O1, P3) within each region. Individual electrode values were treated as repeated measures within each region; no averaging across electrodes was performed prior to feature extraction or statistical analysis. To identify the most sensitive topographical localization of FD changes, we performed a preliminary four-way ANOVA with the topographical factor REGION (anterior-right: F4, C4; posterior-right: P4, O2; anterior-left: F3, C3; posterior-left: P3, O1). Regions that did not show significant Period × Group or Lateralization × Period × Group effects in the initial dispersion analysis, were not the focus of further correlation analysis with the inter-recording interval.

To provide a more detailed analysis of the topographic distribution of the observed differences, we calculated the data for each individual electrode, applying a correction for multiple comparisons. Spearman rank correlation analysis was performed to between neuropsychological scales and the EEG metrics under study and time interval between the EEG recordings. When a significant correlation analysis result was found, an additional analysis of covariates was performed. We also used correlation analysis to evaluate the relationship with neurological and neuropsychological scale scores.

Normality of data distribution was assessed using the Shapiro-Wilk test for age, BMI and dTime. For continuous variables following a normal distribution, independent samples t-tests were used. Categorical variables (sex) were compared using chi-square tests with effect sizes reported as ϕ (phi coefficient).

For within-subject comparisons between the first and second assessment sessions, the Wilcoxon matched pairs test was employed due to the ordinal nature of the neuropsychological scales. Effect sizes were calculated as r = |Z|/√N and are reported alongside the test statistics.

## 3 Results

### 3.1 Dispersion analysis of EEG metrics

**Figure 1:**
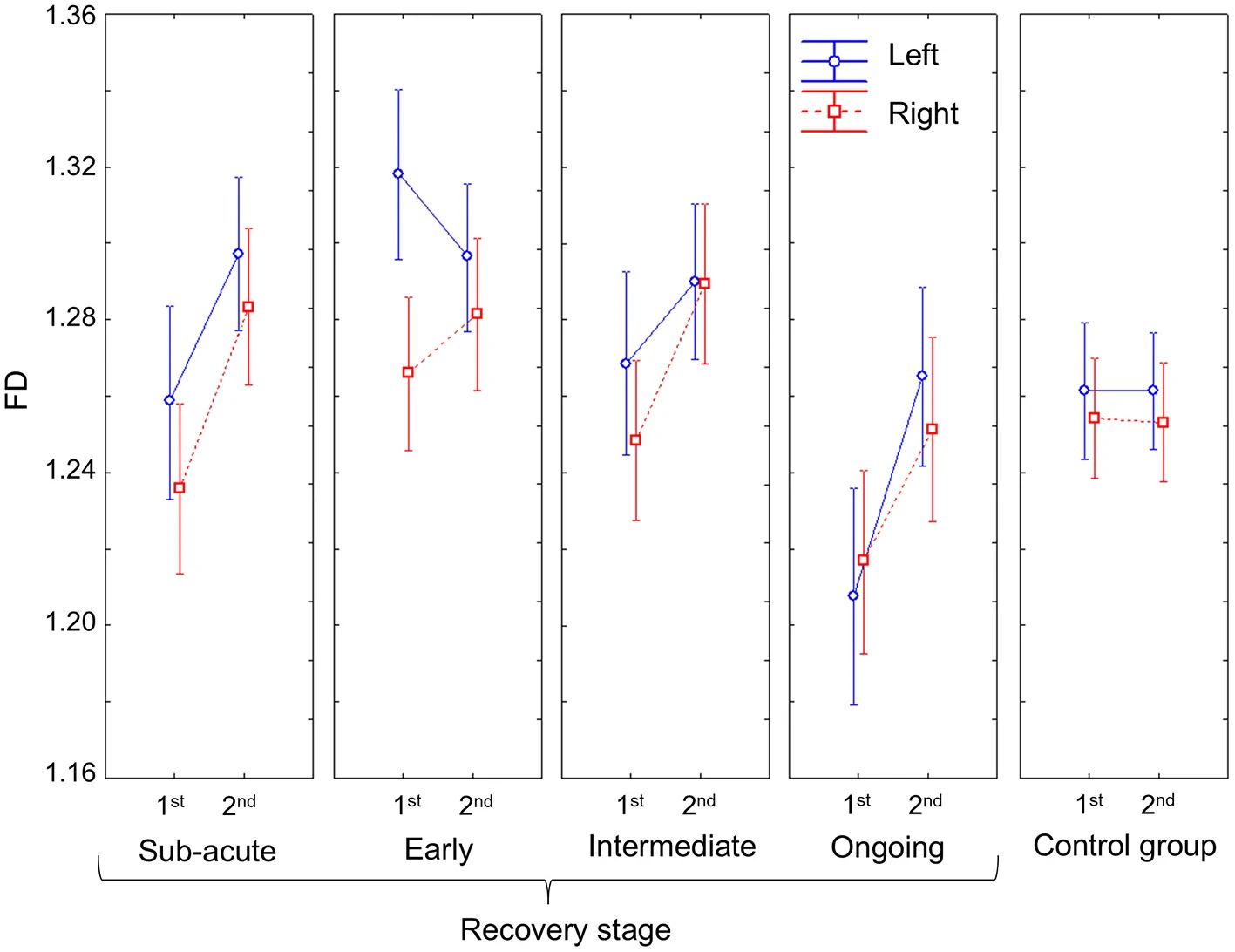
Dynamics of fractal dimension (FD) in the right fronto-central region (electrodes F4 and C4) from the first to the second EEG recording in patients with stroke at different recovery stages and in the control group. Data are presented as mean ± standard deviation. The first recording (1st) was performed before the rehabilitation course, and the second recording (2nd) was performed after its completion. FD values were compared using repeated-measures ANOVA with within-subject factor TIME (1st vs. 2nd) and between-subject factor GROUP (early-stage patients, late-stage patients, healthy controls).

The dynamics of fractal dimension changes demonstrated a relationship with group differences and the lateralization of these differences (Lateralization × Period × Group effect: *F*_(4, 64)_ = 4.73, *p* = 0.008, η^2^_*p*_= 0.17). Specifically, no significant differences were observed between the first and second EEG recordings in healthy volunteers. In contrast, patients exhibited notable dynamics: in all patient groups except those in the early recovery stage, changes were unidirectional in both right and left leads and were characterized by an increase in fractal dimension over the course of rehabilitation. However, in patients in the early recovery stage, a significant decrease in fractal dimension was observed in the left hemisphere, while a significant increase occurred in the right hemisphere in frontal and central areas (see Figure 1). A significant increase in the right hemisphere FD during rehabilitation (from the first to the second time point) was observed across all patient groups, but not in healthy controls (main effect of Period: *F*_(1, 64)_ = 9.44, *p* = 0.003, η^2^_*p*_ = 0.13). Repeated-measures ANOVA revealed that the most significant time-by-group interaction was observed in the right fronto-central region (F4, C4). *Post-hoc* analyses with Bonferroni correction confirmed this increase was significant in patients (*p* < 0.05) but not in controls (*p* = 0.9).

In the left hemisphere, a significant Period × Group interaction was found [*F*_(4, 64)_ = 3.56, *p* = 0.02, η^2^_*p*_ =0.11]. *Post-hoc* tests revealed significant changes in FD for patients in both the ongoing recovery stage (*p* = 0.001) and the early recovery stage (*p* = 0.003). However, the direction of change differed: FD decreased in the left hemisphere for patients in the early recovery stage, whereas it increased for those in the ongoing recovery stage.

Lateralization × Group *F*_(4, 64)_ = 5.5, *p* < 0.001, η^2^_*p*_ = 0.26, Bonferroni correction showed the PAF was significantly higher in the right hemisphere compared to the left only in patients in early and intermediate recovery stage (*p* < 0.01)

**Figure 2:**
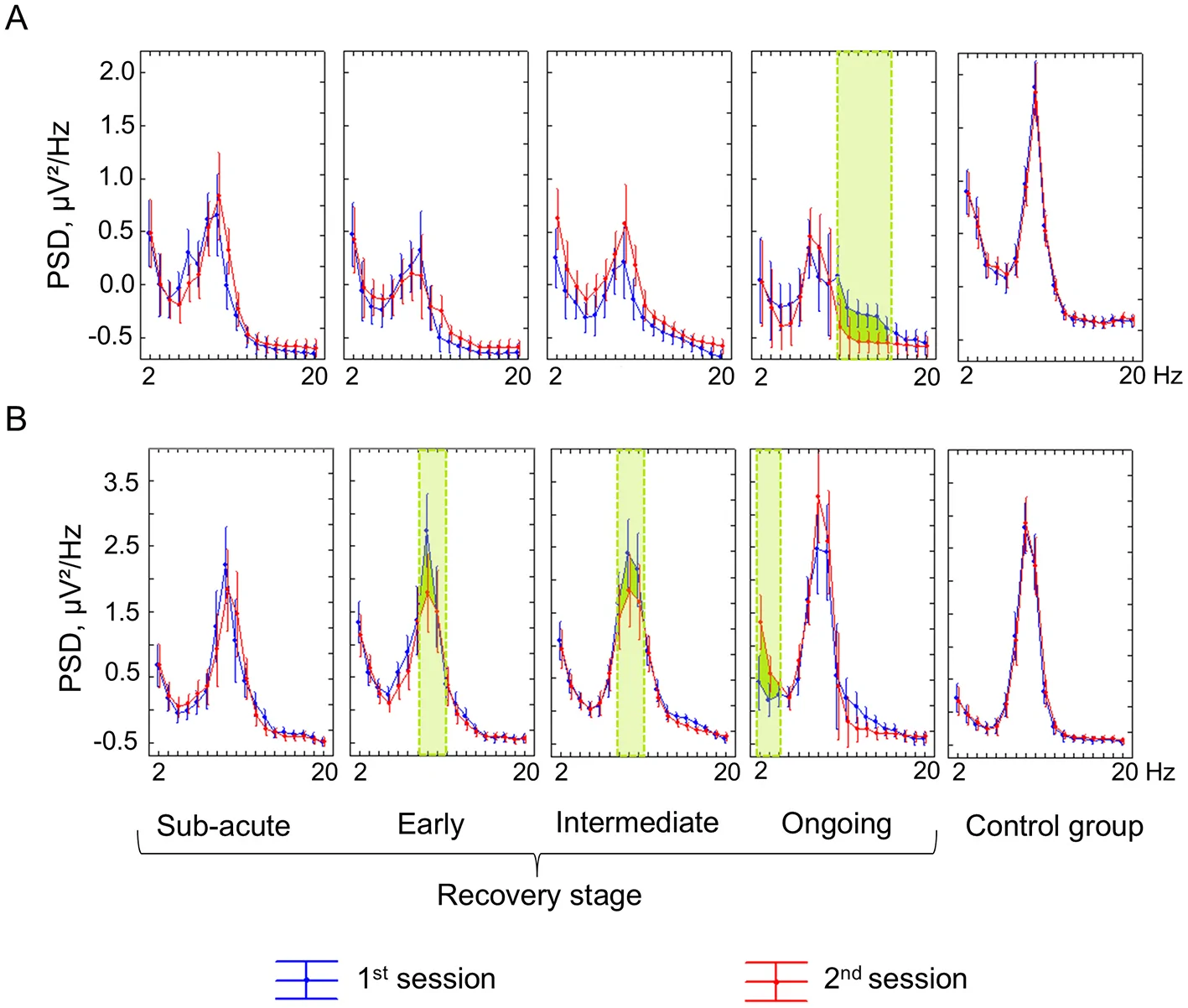
Dynamics of power spectral density (PSD) in the frontal region **(top)** and the right occipito-temporo-parietal region **(bottom)** in stroke patients at different stages of recovery and in the control group. **(A)** Significant differences in frontal leads (F3, Fz, F4); **(B)** Right parietal–occipital leads (O2, P4). Values are presented as mean ± standard deviation. Data were averaged across regions of interest using repeated-measures ANOVA. The dotted line indicates the frequency range in which significant differences were observed.

While the dynamics of EEG PSD changes from the first to the second recording showed no significant differences among healthy volunteers, they differed significantly between stroke patients (at different disease stages) and healthy controls. Significant differences were identified in the frontal leads [Period × Frequency × Group mixed effect: *F*_(68_, _1, 088)_ = 1.46, *p* = 0.01, η^2^_*p*_ = 0.08], which were driven by a significant decrease in beta power (14–20 Hz) specifically in patients in the ongoing recovery stage. A second cluster of differences was found in the right occipito-parietal area [Period × Frequency × Group mixed effect: *F*_(68_, _1, 088)_ = 1.73, *p* < 0.001, η^2^_*p*_ = 0.15], characterized by a significant decrease in the 8–9 Hz alpha rhythm narrow-band PSD in patients at the early and intermediate recovery stages, and a significant increase in 2–6 Hz PSD selectively in patients at the ongoing recovery stage (Bonferroni correction *p* < 0.005). The results are depicted in Figure 2.

### 3.2 Correlation analysis between EEG metrics and EEG sessions time interval

Spearman rank correlation analysis demonstrated that the dynamics of specific EEG parameters were significantly associated with the time elapsed between the first and second recordings. Based on the topographic specificity identified in our preliminary ANOVA (see Methods), we restricted these correlation analyses to the right fronto-central region (F4, C4) for FD and the frontal region (F3, Fz, F4) for beta-rhythm PSD, as other regions did not show significant group-by-time interactions. The change in beta-rhythm PSD in the frontal areas showed a significant negative correlation with the inter-recording interval (*r* = −0.54, *p* < 0.001), meaning it decreased over longer periods. Using ANCOVA we calculated group differences over patients in the dynamics of the beta-rhythm power using inter-recording interval as continuous predictor and found that significant differences in beta-rhythm PSD (previously found in patients in ongoing recovery stage) disappeared (*F* = 4.32, *p* = 0.04, η^2^_*p*_ = 0.1)

**Figure 3:**
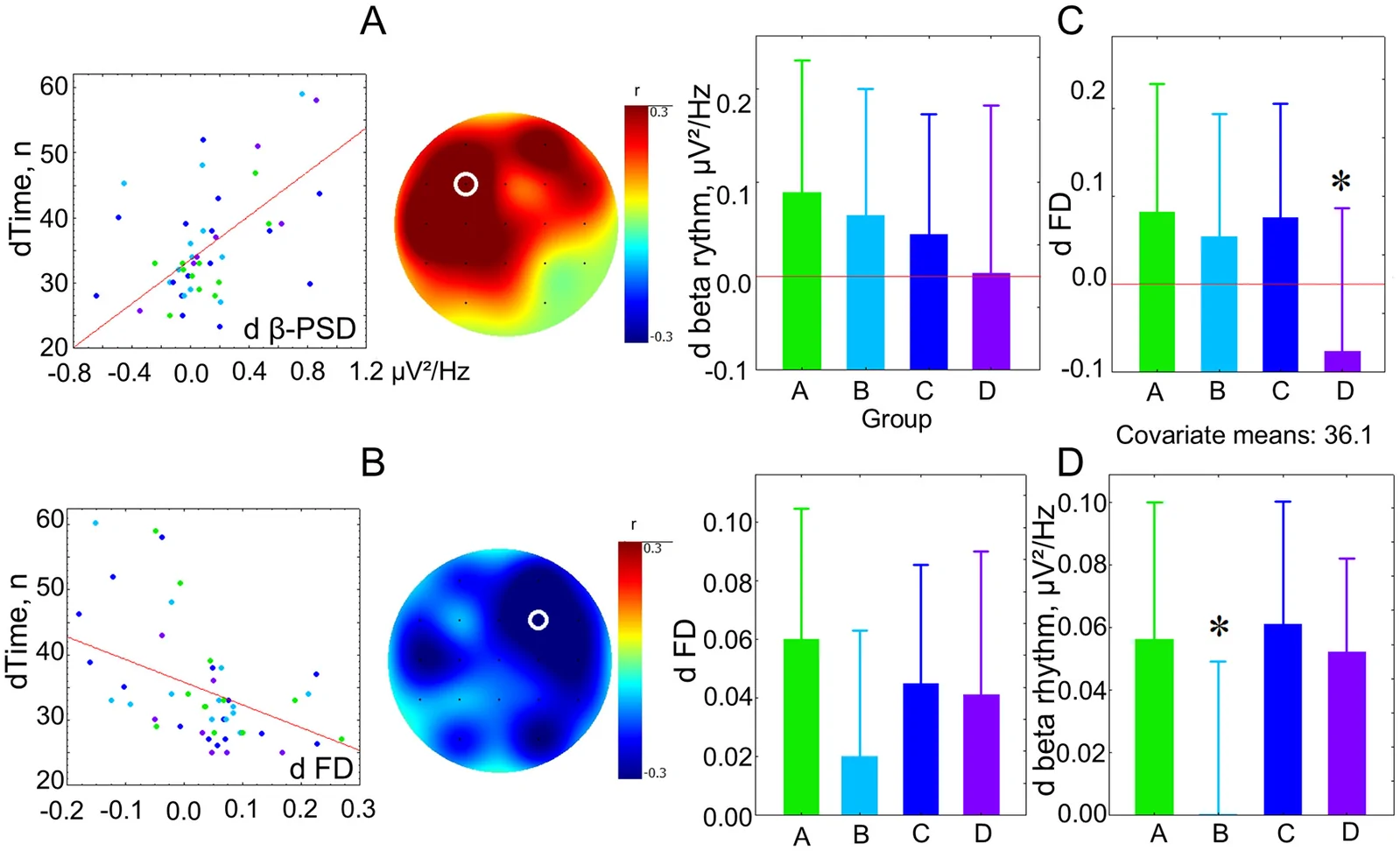
The association between EEG dynamics and the interval between the 1st and 2nd EEG recordings (dTime). **(A)** Beta-rhythm PSD dynamics in the frontal region (F3, Fz, F4). **(B)** FD dynamics in the right fronto-central region (F4, C4). Dots represent individual values for each patient. Colors indicate the groups at different stages of recovery: green—sub-acute group; light blue —early recovery group; blue—intermediate recovery group; violet—ongoing recovery group. **(C, D)** Mean values of EEG dynamics before and after ANCOVA with the inter-recording interval as a continuous predictor. Only regions showing significant group-by-time interactions in the preliminary topographic ANOVA are presented; other regions did not yield significant effects and were therefore not included. The asterisk (*) indicates that, using ANCOVA with the inter-recording interval as a continuous covariate, we found a significant change in correlation values in patients.

Conversely, the change in FD in the right frontal, temporal, and central areas showed a significant positive correlation (*r* = 0.39, *p* = 0.003) with the inter-recording interval, indicating an increase of the FD over time. As was reported, a significant increase in FD in the right hemisphere during rehabilitation was observed across all patient groups. However, using ANCOVA we also calculated group differences over patients in the dynamics of the FD using inter-recording interval as continuous and found that the increase of FD disappeared in patients in early recovery stage (*F* = 8.48, *p* = 0.005, η^2^_*p*_ = 0.18). In the left hemisphere-the effect remain stable (Figure 3).

### 3.3 Correlation analysis between EEG dynamics and dynamics of the rehabilitation scales

**Figure 4:**
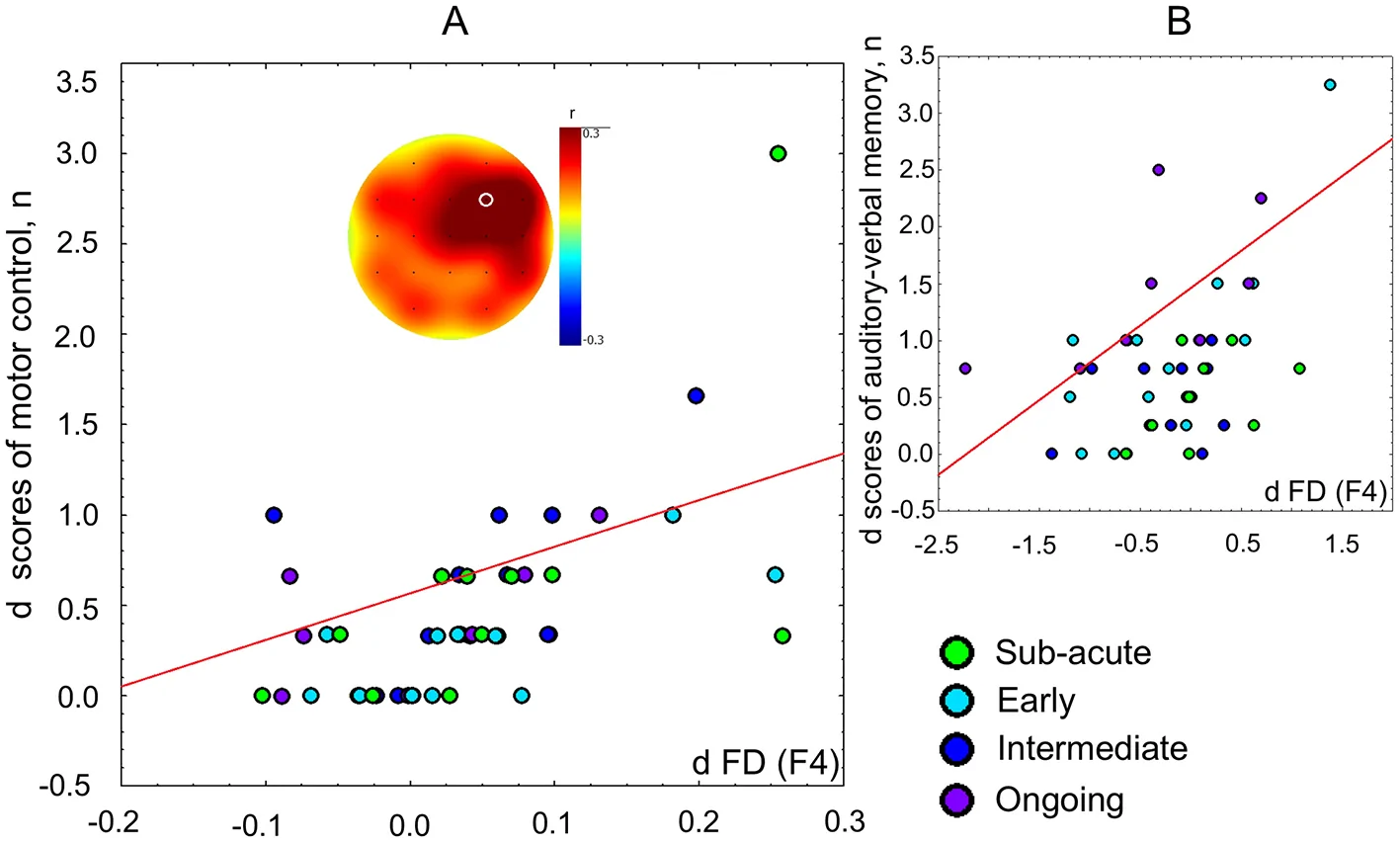
Results of the correlation analysis between EEG dynamics (differences between the 1st and 2nd EEG recordings, denoted as “d”) and the dynamics of the rehabilitation scales (differences between the 1st and 2nd assessments) in patients with stroke at different stages of recovery.

An increase in the fractal dimension over the right hemisphere was significantly correlated with improved motor control function scores (*r* = 0.39, *p* = 0.01). The intra-group correlations remained significant in all sub-groups of patients (*p* < 0.04; Figure 4A).

An increase in slow-wave activity (2–6 Hz) in the right temporo-parieto-occipital region was significantly correlated with improved auditory-verbal memory scores (*r* = 0.52, *p* = 0.001). This correlation was strongest in patients with ongoing recovery (*r* = 0.82, *p* = 0.002) and was also significant in the sub-acute stage (*r* = 0.59, *p* = 0.04) and early recovery (*r* = 0.61, *p* = 0.04) groups, Figure 4B.

A reduction in alpha rhythm power (7–9 Hz) was correlated with improved performance efficiency in motor and motor control functions (*r* = −0.42, *p* = 0.004) and visuospatial ability (*r* = −0.47, *p* = 0.001). Within-group analyses revealed that these correlations were significant only in the early recovery (*r* = −0.8, *p* = 0.002) and intermediate recovery (*r* = −0.68, *p* = 0.01) groups.

## 4 Discussion

Thus, among the indicators associated with the rehabilitation process, an increase in FD over the right hemisphere was observed in all patient groups except for those in the early recovery stage. In this latter group, although a similar right-lateralized increase in FD was present, it was independent of the time interval between EEG recordings. When this temporal factor was controlled for via ANCOVA, the significant differences were no longer evident. This implies that patients in the early recovery stage do not display the typical rehabilitation-related response; rather, the observed increase likely reflects a transition into the intermediate recovery phase. Further underscoring the distinct profile of early recovery, a significant decrease in FD over the left hemisphere was found exclusively in this group and not in the others. An elevated fractal dimension is commonly interpreted as a marker of healthy, adaptable brain function, and its correlation with improvements in praxis aligns with models of motor recovery that emphasize network reorganization. Supporting this, research has demonstrated that the global FD of the EEG signal is significantly reduced in acute stroke patients (4–10 days post-stroke) relative to age-matched healthy controls, indicating a loss of dynamical complexity reflective of global system dysfunction following a focal lesion (Zappasodi et al., 2014). Similarly, both Higuchi FD and signal tortuosity were found to be significantly lower in stroke survivors compared to controls across all post-stroke stages (Rubega et al., 2021).

The observed EEG changes reflect distinct neurophysiological mechanisms that differ between hemispheres and across recovery stages. In the left hemisphere, the early FD decrease over central regions likely arises from local tissue damage, perilesional edema, and secondary degeneration of corticocortical and corticospinal pathways (Carmichael, 2006; Nudo, 2013), reducing signal complexity in damaged networks (Zappasodi et al., 2014). Transcallosal diaschisis—functional depression of regions remote from the lesion—also contributes to bilateral early EEG changes (Carrera and Tononi, 2014; Juhász et al., 1997). The left-central FD decrease we observed exclusively in the early stage thus captures this acute pathological state. In the right hemisphere, loss of transcallosal inhibition from the damaged left hemisphere leads to contralesional disinhibition and hyperexcitability (Nowak et al., 2009; Grefkes and Fink, 2014). This explains the right-hemisphere FD increases across all patient groups, which correlates with motor control gains. The contralesional hemisphere compensates by recruiting alternative pathways, including ipsilateral projections and pre-motor-parietal networks—a process that may be initially maladaptive but becomes beneficial over time (Ward, 2017; Di Pino et al., 2014). Right-central FD increase thus indexes compensatory network dynamics. Recovery depends on restoring interhemispheric balance (Nowak et al., 2009). The early pattern of left FD decrease and right FD increase reflects extreme imbalance; later stabilization of right-hemisphere FD increases alongside functional gains suggests progressive rebalancing. This fits the bimodal balance-recovery model, where moderate contralesional activity is beneficial, but excessive hyperexcitability may be detrimental (Di Pino et al., 2014). The consistent right-hemisphere FD increase across all stages, correlated with praxis gains, supports this view. Stage-specific spectral changes also have clear physiological underpinnings. Early and intermediate alpha desynchronization in occipito-parietal regions reflects cortical disinhibition and reactivation of latent networks (Jensen and Mazaheri, 2010; Ros et al., 2017), consistent with “unmasking” of previously suppressed connections (Jacobs and Donoghue, 1991). In contrast, the late-stage increase in slow-wave activity at the right temporo-parieto-occipital junction, correlated with memory gains, suggests a shift toward synaptic stabilization and homeostatic plasticity (Csercsa et al., 2010; Wilckens et al., 2018). Slow-wave activity promotes synaptic downscaling and consolidation, and its increase in multimodal areas may reflect integration of newly formed networks (Tononi and Cirelli, 2014).

A relationship with the time interval between pre- and post-rehabilitation EEG recordings was also found for the decrease in beta rhythm in the frontal areas. A significant decrease in beta power was observed only in patients in the ongoing recovery stage. However, when an ANCOVA was conducted to control for the influence of the time interval, the significant changes in the ongoing recovery group were no longer present. This suggests that the decrease in beta rhythm in this small subgroup is not a consequence of rehabilitation but is likely associated with other, secondary events.

This study identified three primary, region- and frequency-specific associations between spontaneous EEG dynamics and improvements in neuropsychological rehabilitation scores. First, an increase in fractal dimension over the right hemisphere was linked to gains in motor control function. The positive correlation between right-hemispheric fractal dimension and motor control function suggests that recovery of motor planning and execution may be subserved by a restoration of complex, adaptive neural dynamics in right-sided networks, likely involving fronto-parietal circuits responsible for spatial processing and motor sequencing. Supporting this, FD has been shown to serve as a measure of recovery potential during motor rehabilitation. For example, the Grassberger-Procaccia Fractal Dimension achieved high classification accuracy in distinguishing motor imagery tasks from resting state, and FD-based features have proven effective for patients with impaired sensorimotor rhythms (Liu et al., 2017). Conversely, greater interhemispheric FD asymmetry—specifically, higher FD in the non-lesioned hemisphere during the acute phase—has been correlated with poorer clinical recovery at 6 months, suggesting that FD asymmetry could help identify patients with reduced recovery potential early on, enabling more targeted rehabilitation (Zappasodi et al., 2014). Beyond motor recovery, FD also enhances motor imagery BCI classification by capturing non-linear signal complexity, particularly when multiple FD methods are integrated. This approach holds strong potential for improving intention detection and rehabilitation assessment (Mohamed and Jusas, 2025). Furthermore, FD has demonstrated relevance in broader neurobehavioral contexts: intra-subject increases in Higuchi FD have been observed even during spontaneous recovery without structured rehabilitation (Rubega et al., 2021), and EEG fractal dimensions have been shown to predict high-level behavioral responses in patients with minimal consciousness (Liuzzi et al., 2023).

Second, we found, that increased right-hemispheric slow-wave (2–6 Hz) activity in temporo-parieto-occipital regions was correlated with improvements in auditory-verbal memory. A notable finding is the strong correlation between heightened 2–6 Hz activity in the right temporo-parieto-occipital cortex and enhanced auditory-verbal memory performance. We propose that this increase in right-sided slow-wave activity may not reflect pathological slowing, but rather could serve as a potential marker of active neuroplasticity or cortical idling in language-related regions. While slow waves are conventionally linked to drowsiness or pathology, in a rehabilitation context they may instead reflect targeted, activity-dependent neuroplastic processes—such as synaptic pruning or memory consolidation—particularly in the right-hemisphere homologs of the classic left-hemisphere language areas that were impaired in our patients with left-hemisphere stroke. Previous research supports a distinct role for slow-wave activity in the damaged hemisphere: for instance, delta activity with a dipole localized near the structural lesion has been negatively associated with language improvement (Hensel et al., 2004). In that study, language function improved significantly during the first year post-stroke, paralleling a reduction in left-hemispheric delta activity. This suggests that slow-wave activity in the right hemisphere may have a different, potentially facilitatory role in recovery. Indeed, the pronounced right-hemispheric slow-wave activity observed in the “ongoing recovery” group supports the interpretation of an active, plastic state conducive to memory reconsolidation. A review by Wilckens (Wilckens et al., 2018) indicates that enhanced slow-wave activity can be associated with improved cognitive function, achievable through cognitive training, intensive learning, meditation, and transcranial stimulation. Such interventions can lead to local increases in slow-wave activity within the cortical regions engaged during training, along with corresponding task-specific performance gains. Furthermore, in spinal cord injury rehabilitation, low-frequency EEG oscillations have been identified as potential biomarkers of motor recovery and psychological status, suggesting a salutogenic compensatory mechanism within the central nervous system. These associations are modulated by factors including cognitive-emotional status, neurological impairment, lesion duration and level, pain sensitivity, and cortical excitability parameters (Lacerda et al., 2024).

A reduction in alpha power was associated with enhanced performance in motor activity, motor control functions and tasks related to visuospatial abilities. This inverse correlation aligns with the established role of alpha rhythms in cortical inhibition, where a power decrease signifies a state of cortical disinhibition or heightened readiness, facilitating neural resource allocation. This phenomenon appears context-dependent. For instance, in patients with mild traumatic brain injury, excessive frontal alpha desynchronization during cognitive tasks immediately post-injury is interpreted as a marker of compensatory effort and incomplete neural recovery, reflecting lower cognitive efficiency (Arakaki et al., 2018). Conversely, studies of elite athletes demonstrate the opposite pattern within their domain of expertise: during skilled movements, athletes exhibit a weaker alpha desynchronization (i.e., a smaller power decrease) in primary motor and pre-motor areas compared to non-athletes (Del Percio et al., 2010). This attenuated desynchronization is a signature of “neural efficiency,” indicating that experts require less cortical activation for optimal performance. Crucially, this effect is skill-specific, as no such difference was observed when athletes observed unfamiliar motor acts (Del Percio et al., 2010). Synthesizing these findings, a reduction in alpha power may represent a common neural mechanism of active, focused engagement. In the context of recovery from injury or the re-learning of skills, it likely reflects compensatory cortical recruitment to overcome deficit. In the context of expertise, it reflects efficient, streamlined processing. The observation that this correlation is specific to the early and intermediate stages of recovery supports the interpretation that it is a hallmark of an active re-learning phase, where a state of reduced cortical inhibition benefits the integration and performance of relearned tasks.

We observed that neural correlates of recovery were not constant but shifted across different phases. Early- and mid-stage recovery were characterized by a specific link between reduced alpha power and improved performance. In contrast, during ongoing recovery, a connection between slow-wave activity and memory emerged as the strongest predictor. This pattern points to a dynamic, stage-specific process of neuroplastic remodeling. This aligns with established literature highlighting that the brain's capacity for repair and the optimal rehabilitation strategy differ fundamentally across the acute, subacute, and chronic phases of stroke recovery, with the effectiveness of an intervention depending critically on its timing and the patient's specific neural state (Yan et al., 2025). The literature delineates a phased progression, in particular, in the acute phase, the contralesional (healthy) hemisphere often shows heightened activity, a state frequently correlated with poorer long-term outcomes and linked to a breakdown in interhemispheric communication (Alia et al., 2017). The subacute phase represents a critical window of heightened plasticity, where the focus of recovery shifts to reorganization within the perilesional cortex of the damaged hemisphere, whereas during the chronic phase, the focus shifts to consolidating and maintaining gains through long-term rehabilitation, which sustains neurotrophic signaling and supports the integration of newly formed circuits (Alia et al., 2017; Yan et al., 2025). Our results imply that the neural substrates of recovery shift dynamically: early stages may involve cortical disinhibition and the re-activation of latent networks, while later stages engage distinct mechanisms, such as slow-wave mediated systems consolidation. Consequently, rehabilitation strategies may need to be precisely timed and individually tailored to target these specific, evolving neurophysiological states.

Our results also revealed specific regional dynamics that evolved over the course of rehabilitation, consistent with the concept of hierarchical reorganization (Ward, 2017), whereby distant and homotopic regions are recruited sequentially depending on the stage and severity of the lesion. This supports the view that recovery unfolds as a spatiotemporal cascade. In the central region, right-hemisphere FD increases correlated with motor control gains, reflecting compensatory engagement of contralesional sensorimotor networks (Zappasodi et al., 2014; Rubega et al., 2021), whereas the early-stage left-central FD decrease likely indicates perilesional dysfunction or diaschisis (Juhász et al., 1997). In the frontal region, the time-dependent decrease in beta rhythm suggests gradual normalization of prefrontal oscillatory dynamics, which may facilitate cognitive control and top-down modulation; beta-band activity is thought to sustain the current sensorimotor or cognitive set, and its pathological increase may impair flexible behavioral control (Engel and Fries, 2010). The parietal and occipital regions showed distinct spectral patterns: alpha power reductions in occipito-parietal areas correlated with visuospatial and motor improvements, specifically in the early and intermediate stages, suggesting that cortical disinhibition enhances spatial processing during the critical window of plasticity (Ros et al., 2017). In addition, the increase in slow-wave activity at the right temporo-parieto-occipital junction, which correlated with auditory-verbal memory gains, points to a compensatory role for multimodal association areas in memory consolidation; slow-wave activity enhancement has been linked to synaptic consolidation and cognitive improvement (Mouthon et al., 2021; Csercsa et al., 2010). Importantly, these regional patterns shifted across stages: early reorganization was dominated by left-central FD decrease and right-fronto-central FD increase, intermediate stages featured occipito-parietal alpha desynchronization linked to visuospatial gains, and ongoing recovery involved right-TPO slow-wave increases associated with memory consolidation. This stage-dependent regional profile is consistent with phased neuroplasticity models (Alia et al., 2017; Yan et al., 2025) and underscores that rehabilitation timing should be aligned with the dominant regional processes at each stage—early interventions aimed at preserving left-central integrity and enhancing right-central excitability, intermediate interventions focused on modulating occipito-parietal alpha oscillations, and later interventions targeting right-TPO slow-wave activity to support memory consolidation (Sirpal et al., 2025; Mark et al., 2024). Taken together, these findings indicate that post-stroke reorganization is not a uniform process but involves stage-specific, regionally distributed neuroplastic changes that complement the broader hemispheric compensation framework and may inform targeted neuromodulation strategies.

These results have direct implications for neurorehabilitation. First, they provide candidate EEG biomarkers for objectively monitoring recovery trajectories in specific cognitive domains. Second, they support the potential for neuromodulation therapies to be precisely targeted—for instance, enhancing right-hemispheric complexity or modulating alpha rhythms—to potentiate recovery of praxis or memory. Theoretically, the data reinforce a model of recovery as a series of distinct neurophysiological phases rather than a linear process.

### 4.1 Limitations and future directions

Several limitations must be considered. The sample sizes within sub-groups, while sufficient for detecting correlations, warrant replication in larger cohorts.

### 4.2 Conclusions

FD emerges as a potent and sensitive biomarker, capturing the restoration of adaptive, complex neural dynamics. The rehabilitation-associated increase in FD over the right hemisphere, correlated with kinetic praxis gains, underscores the compensatory engagement of non-lesioned networks. The unique decrease in left-hemisphere FD observed exclusively in the early recovery stage may signify a distinct, transient state of pathological disorganization or active rewiring preceding stabilization.

Recovery is not a monolithic process but comprises distinct neurophysiological phases. Early and intermediate stages are characterized by alpha-band desynchronization linked to praxis improvement, suggesting a mechanism of cortical disinhibition for network reactivation. Later, “ongoing” stages involve increased right-hemispheric slow-wave activity associated with memory consolidation, pointing toward a shift toward neuroplastic integration and synaptic homeostasis.

The consistent finding of right-hemispheric EEG changes correlating with functional improvements highlights the brain's capacity for inter-hemispheric reorganization. This reinforces the concept that the contralesional hemisphere is not merely a passive observer but an active participant in functional recovery, particularly for domains like motor planning and verbal memory.

The identified EEG signatures—FD, posterior slow-wave power, and frontal alpha/beta rhythms-offer a quantifiable, multi-dimensional biomarker toolkit. These metrics can move rehabilitation beyond generalized protocols toward a physiology-informed, personalized model targering to enhance right-hemispheric complexity in patients with motor planning deficits or to modulate specific spectral bands based on the patient's recovery stage.

## Data Availability

The data analyzed in this study is subject to the following licenses/restrictions: the data are available from the corresponding authors upon reasonable request. Requests to access these datasets should be directed to Larisa Mayorova, larimayor@gmail.com; Galina Portnova, caviter@list.ru.
